# Mitochondrial D NA Analysis from Exome Sequencing Data Improves Diagnostic Yield in Neurological Diseases

**DOI:** 10.1002/ana.26063

**Published:** 2021-04-01

**Authors:** Olivia V. Poole, Chiara Pizzamiglio, David Murphy, Micol Falabella, William L. Macken, Enrico Bugiardini, Cathy E. Woodward, Robyn Labrum, Stephanie Efthymiou, Vincenzo Salpietro, Viorica Chelban, Rauan Kaiyrzhanov, Reza Maroofian, the SYNaPS Study Group, Issam Alkhawaja, Selina Banu, Maria Bonsignore, Marianthi Breza, Gabriella Di Rosa, Morteza Heidari, Georgios Koutsis, Arn M.J.M. van den Maagdenberg, Alfons Macaya, Alexander Münchau, Carmela Scuderi, Nazira Zharkinbekova, Anthony A. Amato, Allison Gregory, Susan J. Hayflick, Queen Square Genomics, Hallgeir Jonvik, Nicholas Wood, Henry Houlden, Jana Vandrovcova, Michael G. Hanna, Alan Pittman, Robert D.S. Pitceathly

**Affiliations:** ^1^ Department of Neuromuscular Diseases UCL Queen Square Institute of Neurology and The National Hospital for Neurology and Neurosurgery London UK; ^2^ Department of Clinical and Movement Neurosciences UCL Queen Square Institute of Neurology and The National Hospital for Neurology and Neurosurgery London UK; ^3^ Department of Neuromuscular Diseases UCL Queen Square Institute of Neurology London UK; ^4^ Neurogenetics Unit The National Hospital for Neurology and Neurosurgery London UK; ^5^ Department of Neurology Brigham and Women's Hospital and Harvard Medical School Boston MA; ^6^ Departments of Molecular and Medical Genetics, Pediatrics, and Neurology Oregon Health and Science University Portland OR; ^7^ UCL Queen Square Institute of Neurology London UK; ^8^ Genetics Research Centre St. George's, University of London London UK

## Abstract

A rapidly expanding catalog of neurogenetic disorders has encouraged a diagnostic shift towards early clinical whole exome sequencing (WES). Adult primary mitochondrial diseases (PMDs) frequently exhibit neurological manifestations that overlap with other nervous system disorders. However, mitochondrial DNA (mtDNA) is not routinely analyzed in standard clinical WES bioinformatic pipelines. We reanalyzed 11,424 exomes, enriched with neurological diseases, for pathogenic mtDNA variants. Twenty‐four different mtDNA mutations were detected in 64 exomes, 11 of which were considered disease causing based on the associated clinical phenotypes. These findings highlight the diagnostic uplifts gained by analyzing mtDNA from WES data in neurological diseases. ANN NEUROL 2021;89:1240–1247

The rapid expansion in recognized inherited neurological disorders has led to a “genetics first” approach to their diagnosis, which frequently involves the early application of clinical whole exome sequencing (WES).^1^ There is also growing evidence that the iterative re‐analysis of exome data significantly improves the diagnostic yield of pathogenic DNA variants in rare diseases, and should therefore be considered in all unresolved cases.^2^

Primary mitochondrial diseases (PMDs) have an adult prevalence of 1 in 4,300,^3^ thus represent some of the most common neurogenetic disorders, and frequently manifest with nervous system phenotypes that overlap with other neurological disorders. More than 300 mitochondrial proteins, encoded by both nuclear and mitochondrial genomes, are linked with PMDs^4^; however, mitochondrial DNA (mtDNA) mutations account for the vast majority in adults.^3^ Importantly, although mtDNA reads are generated during WES, they are not routinely analyzed. Consequently, standard clinical WES bioinformatic pipelines do not report disease causing mtDNA variants.

We applied a Genome Analysis Toolkit (GATK) Mutect2‐based^5^, ^6^ bioinformatics pipeline to analyze the mtDNA reads from 11,424 exomes, generated by University College London Queen Square Genomics Facility, and determined the diagnostic uplifts gained by including mtDNA analysis during the iterative reanalysis of neurological disease exomes.

## Methods

This study was approved by the Queen Square Research Ethics Committee, London, UK (09/H0716/76).

### Ascertainment of Whole Exome Sequencing Data and Cohort Phenotypic Groups

In total, 11,424 DNA samples, extracted from blood, which had undergone WES analysis at the University College London Queen Square Genomics Facility between 2011 and 2019, were included in the study. The cohort was composed of probands with a range of neurological phenotypes, their relatives, and healthy controls. The most common clinical group comprised extrapyramidal and movement disorders (International Classification of Disease‐10 code G20–G26^7^; 31.5%), followed by systemic atrophies, primarily affecting the central nervous system (G10–G14; 17%), episodic and paroxysmal disorders (G40–G47; 16.4%), diseases of myoneural junction and muscle (G70–G73; 7.5%), other degenerative diseases of the nervous system (G30–G32; 7.4%), polyneuropathies and other disorders of the peripheral nervous system (G60–G64; 3.6%), and mitochondrial metabolism disorders (E88.4; 1.4%). Neurologically normal controls accounted for 15.2% of the exomes.

### Mitochondrial Variant Calling and Filtering

**FIGURE 1:**
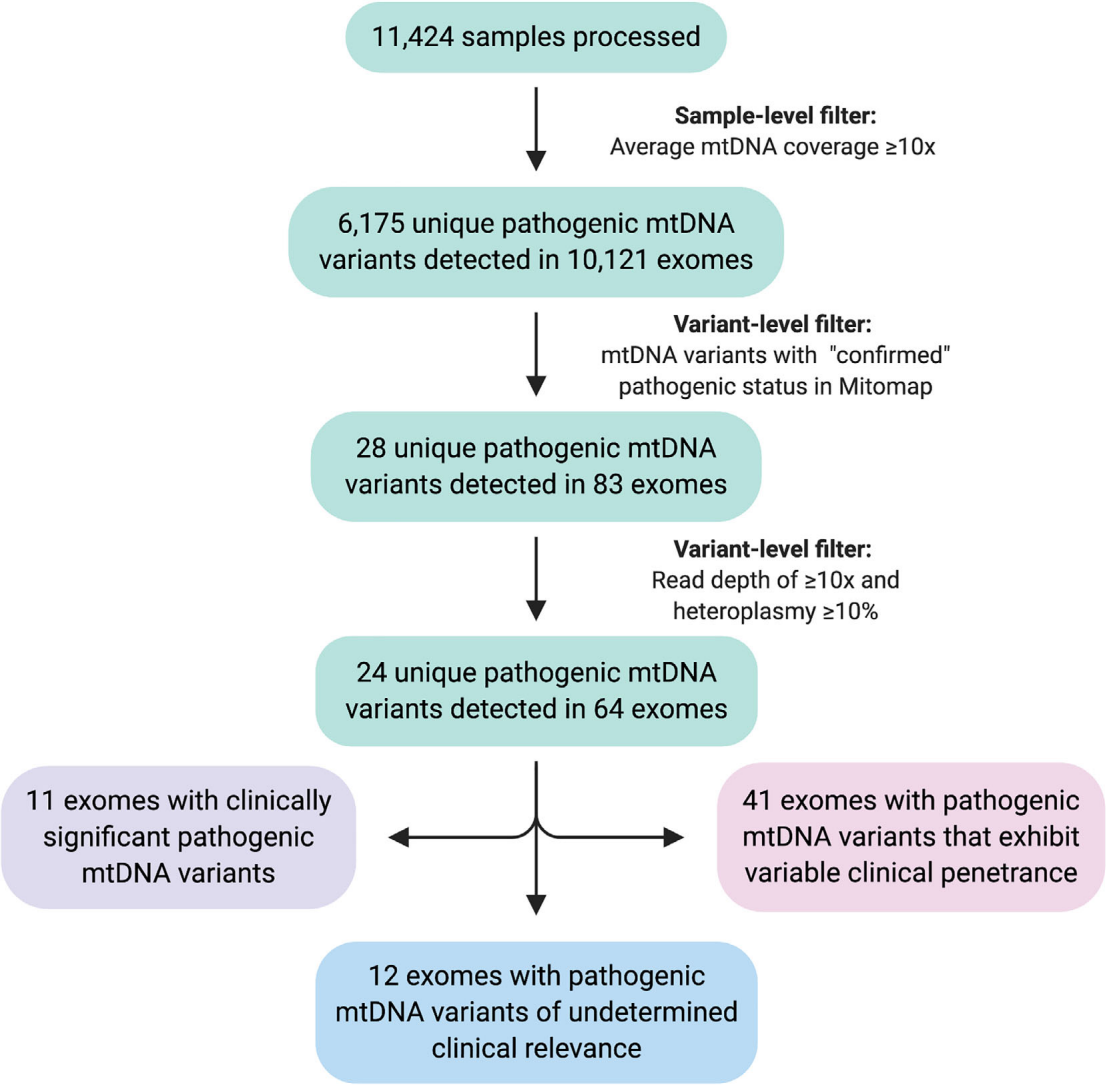
Flow diagram summarizing sample‐level and variant‐level filtering steps applied to 11,424 samples that underwent exome sequencing analysis at the University College London Queen Square Genomics Facility between 2011 and 2019. The figure was created using BioRender.com. mtDNA, mitochondrial DNA. [Color figure can be viewed at www.annalsofneurology.org]

Exome capture libraries were generated using SureSelect (Agilent), Nextera (Illumina) or TrueSeq (Illumina) kits, and all samples were sequenced using Illumina instruments generating 100 to 150 bp paired‐end reads. Fastq files were aligned to the GRCh38 genome reference, which includes the revised Cambridge Reference Sequence (rCRS) of human mtDNA, using NovoAlign version 3.08.02 (Novocraft). Alignments were further processed according to the GATK version 4 best practices^5^ and variants in mtDNA were called using a somatic variant caller, Mutect2,^6^ in tumor‐only mode and filtered using Filter Mutect Calls tool. Only variants in exomes with an average mtDNA coverage of ≥10 times were kept for downstream analysis (Fig 1) and annotated using Variant Effect Predictor,^8^ Mitomap,^9^ and an in‐house database of mtDNA variants. Variants of interest were defined as those with a “confirmed” pathogenic status in Mitomap. Variants with a read depth ≥10 times and heteroplasmy level of ≥10% in blood were validated and selected for further clinical evaluation.

### Confirmation of Pathogenic Mitochondrial DNA Variants

All pathogenic mtDNA variants identified from WES data were subsequently confirmed using whole mtDNA next generation sequencing (NGS). Long‐range polymerase chain reaction (PCR) amplification of 2 overlapping fragments covering the entire mtDNA preceded NGS on the MiSeq platform using the Illumina NextEra XT library preparation. Data analysis was performed using an in‐house pipeline aligned to the rCRS, GenBank accession number NC_012920.1. Coverage of the coding region (nucleotides 577–16023) was at a minimum depth of 1,000 times. Variant detection sensitivity was greater than 95% (95% confidence interval).

### Classification of Pathogenic Mitochondrial DNA Variants

Pathogenic mtDNA variants were stratified according to their associated neurological presentations as follows: (1) clinically significant pathogenic mtDNA variants, defined as a well‐recognized/reported clinical phenotype and a mutant mtDNA heteroplasmy level of ≥10% in blood; (2) pathogenic mtDNA variants of undetermined clinical relevance, defined as an atypical clinical phenotype and a mutant mtDNA heteroplasmy level of ≥10% in blood; and (3) pathogenic mtDNA variants that exhibit variable clinical penetrance at near homoplasmic or homoplasmic mutant levels and a mutant mtDNA heteroplasmy level of ≥10% in blood (Supplementary Table S1 summarizes a list of pathogenic mtDNA variants that exhibit this phenomenon).

## Results

In total, 6,175 mitochondrial variants were identified across 10,121 exomes with sufficient mtDNA coverage (median mtDNA coverage = 43.9 times; see Fig 1). This included 28 pathogenic mtDNA variants in 83 exomes. Twenty‐four variants in 64 exomes met our threshold for further clinical evaluation; specifically, a locus‐specific depth ≥10 times and a heteroplasmy level ≥10% (Tables 1, 2, 3 and Supplementary Table S2).

**TABLE 1:** Clinically Significant Pathogenic Mitochondrial DNA Variants Detected in the 11,424 Exomes Analyzed

Subject	mtDNA variant	Gene	No. ref reads	No. alt reads	Het (%)	Reported associated disease^9^
1	m.3697G>A	*MT‐ND1*	7	89	92	MELAS/LS/LDYT
2	m.8851T>C	*MT‐ATP6*	1	61	98	BSN/LS
3	m.8993T>C	*MT‐ATP6*	0	65	98	NARP/LS/MILS/other
4	m.9185T>C	*MT‐ATP6*	1	76	97	LS/Ataxia syndromes/NARP‐like disease
5	m.9185T>C	*MT‐ATP6*	7	96	92	LS/Ataxia syndromes/NARP‐like disease
6	m.10158T>C	*MT‐ND3*	19	6	25	LS/MELAS
7	m.13094T>C	*MT‐ND5*	87	55	39	Ataxia and PEO/MELAS, LS, myoclonus, fatigue
8	m.13513G>A	*MT‐ND5*	47	54	53	LS/MELAS/LHON‐MELAS overlap syndrome
9	m.13513G>A	*MT‐ND5*	19	3	14	LS/MELAS/LHON‐MELAS overlap syndrome
10	m.14459G>A	*MT‐ND6*	1	83	96	LDYT/LS
11	m.14459G>A	*MT‐ND6*	2	78	97	LDYT/LS

BSN = bilateral striatal necrosis; Het = heteroplasmy; LDYT = Leber hereditary optic neuropathy and dystonia; LHON = Leber hereditary optic neuropathy; LS = Leigh syndrome; MELAS = mitochondrial encephalomyopathy with lactic acidosis and stroke‐like episodes; MILS = maternally inherited Leigh syndrome; mtDNA = mitochondrial DNA; NARP = neurogenic muscle weakness, ataxia, and retinitis pigmentosa; PEO = progressive external ophthalmoplegia.

**TABLE 2:** Clinical Features of the Patients with Clinically Significant Pathogenic Mitochondrial DNA Variants in the 11,424 Exomes Analyzed

Subject	Age, yr/sex	Subject phenotype	Muscle pathology and RCEA	Brain MRI	Family history
1	3/M	Encephalopathy, lower limb spasticity, generalized tonic–clonic seizures, palatal myoclonus	NA	NA	NA
2	82/M Deceased	Progressive ataxia (onset in 50s), cognitive impairment	NA	Mild supratentorial atrophy with disproportionate cerebellar atrophy and sparing of the brainstem	Identical twin brother with ataxia
3	13/M	Developmental delay, spastic‐dystonic gait, ID, axonal neuropathy	NA	NA	One older sibling with developmental delay and ID
4	45/M Deceased	Young adult onset progressive cerebellar ataxia, tremor, optic atrophy, neuropathy, myoclonic epilepsy, urinary frequency, atrial fibrillation	RRF	Basal ganglia changes and cerebellar atrophy	One affected brother (subject 5), mother asymptomatic (47% mutant load in blood), maternal grandmother with epilepsy
5	49/M	Young adult onset progressive cerebellar ataxia, pyramidal signs, neuropathy, restless leg syndrome, ADHD	NA	Marked cerebellar atrophy	Less severely affected brother of subject 4
6	29/F	Stroke‐like episodes, cerebellar ataxia, left sided spastic hemiparesis, learning difficulties, seizures, optic atrophy, mild scoliosis, incomplete RBBB	Mild increase in lipid, no RRF or COX negative fibers Low complex I	White matter changes and high signal in right basal ganglia and right frontoparietal deep white matter	Mother asymptomatic
7	27/M	Encephalopathic episode with bilateral INO and diplopia, nocturnal hypoventilation, reduced visual acuity, LVH	Normal pathology Normal RCEA	Symmetrical midbrain, pontine tegmental, brainstem and peduncular T2 signal hyperintensities	Mother and 2 brothers asymptomatic
8	26/F	LS, learning disability, limb dystonia, regression of mobility and speech after the age of 9 yr, raised CSF lactate	NA	Basal ganglia lesions	Mother asymptomatic
9	20/F Deceased	Focal epilepsy, mild ID, failure to thrive, dysphagia, constipation, respiratory insufficiency with tracheostomy	RRF Normal RCEA	Bilateral and symmetrical T2 high signal in basal ganglia, white matter changes in the periventricular frontal region	NA
10	52/F	Cognitive abnormalities, slowly progressive gait changes with onset at 30 yr	NA	NA	One affected brother (subject 11)
11	45/M Deceased	Gait changes with onset at 20 yr	NA	NA	One affected sister (subject 10)

ADHD = attention deficit hyperactivity disorder; COX = cytochrome *c* oxidase; CSF = cerebrospinal fluid; ID = intellectual disability; INO = intra‐nuclear ophthalmoplegia; LS = Leigh syndrome; LVH = left ventricular hypertrophy; MRI = magnetic resonance imaging; NA = not available; RBBB = right bundle branch block; RCEA = respiratory chain enzyme activity; RRF = ragged red fibers.

**TABLE 3:** Pathogenic Mitochondrial DNA Variants of Undetermined Clinical Relevance in the 11,424 Exomes Analyzed

Subject	Age, yr /sex	mtDNA variant	Gene	No. ref reads	No. alt reads	Het (%)	Reported associated disease^9^	Subject phenotype/ investigations
12	77/F	m.3243A>G	*MT‐TL1*	0	17	98	MELAS/LS/DMDF/MIDD/SNHL/CPEO/MM/FSGS/ASD/cardiac + multi‐organ dysfunction	IBM diagnosed 2012 Onset of symptoms aged 40 yr with grasp and proximal lower limb weakness Muscle biopsy; endomysial inflammatory cell infiltrate with invasion of non‐necrotic muscle fibers, fibers with rimmed vacuoles, ragged red and COX negative fibers
13	NA	m.3243A>G	*MT‐TL1*	10	4	29	MELAS/LS/DMDF/MIDD/SNHL/CPEO/MM/FSGS/ASD/Cardiac and multi‐organ dysfunction	Adult subject with brain calcifications, no family history
14	3/f	m.3260A>G	*MT‐TL1*	7	16	68	MMC/MELAS	Hypotonia since birth, severe intellectual disability, stereotyped hand movements, choreic movements, dystonic postures, anarthria MRI; thin corpus callosum with delayed myelination Confirmed heterozygous *VAMP2* mutation
15	NA	m.3271T>C	*MT‐TL1*	51	20	30	MELAS/DM	MSA; pathologically confirmed
16	NA	m.7497G>A	*MT‐TS1*	32	26	45	MM/EXIT	MSA; pathologically confirmed
17	NA	m.8344A>G	*MT‐TK*	54	107	65	MERRF; Other ‐ LS/depressive mood disorder/Leukoencephalopathy/HCM	MSA; pathologically confirmed
18	NA	m.8851T>C	*MT‐ATP6*	22	5	21	BSN/LS	Unaffected subject
19	NA	m.8993T>G	*MT‐ATP6*	20	28	58	NARP/Leigh Disease/MILS/Other	PD
20	NA	m.9176T>C	*MT‐ATP6*	17	15	47	FBSN/Leigh disease	Unaffected subject
21	NA	m.9185T>C	*MT‐ATP6*	16	5	25	Leigh disease/Ataxia syndromes/NARP‐like disease	Extrapyramidal disorder
22	NA	m.11778G>A	*MT‐ND4*	4	11	73	LHON/progressive dystonia	PD
23	21/m	m.13513G>A	*MT‐ND5*	69	27	28	Leigh disease/MELAS/LHON‐MELAS overlap syndrome	Cyclic vomiting syndrome

ASD = autistic spectrum disorder; BSN = bilateral striatal necrosis; CPEO = chronic progressive external ophthalmoplegia; DM = diabetes mellitus; DMDF = diabetes deafness; EXIT = exercise intolerance; FBSN = familial bilateral striatal necrosis; FSGS = focal segmental glomerulosclerosis; HCM = hypertrophic cardiomyopathy; Het = heteroplasmy; IBM = inclusion body myositis; LHON = Leber hereditary optic neuropathy; LDYT = LHON and dystonia; LS = Leigh syndrome; MELAS = mitochondrial encephalomyopathy with lactic acidosis and stroke‐like episodes; MERRF = Myoclonic epilepsy with ragged‐red fibers; MIDD = Maternally inherited diabetes and deafness; MILS = maternally inherited Leigh syndrome; MM = mitochondrial myopathy; MMC = maternal myopathy and cardiomyopathy; MSA = multi‐system atrophy; mtDNA = mitochondrial DNA; NA = not available; NARP = neurogenic muscle weakness, ataxia, and retinitis pigmentosa; No. = number; PD = Parkinson's disease; SNHL = sensorineural hearing loss.

### Clinically Significant Pathogenic Mitochondrial DNA Variants

Clinically significant pathogenic mtDNA variants were detected in 11 exomes from 9 unrelated families (mean age = 36 years; age range = 3–82 years; 36% female; see Tables 1 and 2). The variants broadly resided within 2 groups of genes: 7 pathogenic variants were detected in mitochondrial complex I subunit genes (*MT‐ND1*, *MT‐ND3*, *MT‐ND5*, and *MT‐ND6*); and 4 pathogenic variants resided in *MT‐ATP6*, encoding the ATP6 subunit of mitochondrial ATP synthase (complex V). Mutant levels of the *MT‐ATP6* variants were uniformly high in blood (92–98%). This corresponded to predominantly central nervous system (CNS) phenotypes, including Leigh syndrome (LS) and Leigh‐like syndrome, which typically occur when the mutant load is ≥90%.^10^ One individual survived into the 9th decade despite harboring 98% m.8851T>C levels, a mutant load previously linked with severe pediatric phenotypes.^11^, ^12^ Mutant mtDNA levels in exomes with pathogenic complex I subunit variants were more variable in blood (14–97%). There was also a less obvious correlation between the clinical phenotype and the underlying mutant load, given that all patients had severe CNS phenotypes (LS/Leigh‐like syndrome and mitochondrial encephalomyopathy, lactic acidosis and stroke‐like episodes [MELAS]), despite some harboring relatively low heteroplasmy levels in blood. Variable neurological manifestations have previously been reported with low mutant *MT‐ND5* levels,^13^ thus reaffirming that pathogenic mtDNA variants measured peripherally (even in post‐mitotic tissues) do not always accurately reflect CNS levels.

### Pathogenic Mitochondrial DNA Variants of Undetermined Clinical Relevance

Pathogenic mtDNA variants with undetermined clinical relevance were detected in 12 exomes from 12 unrelated families (see Table 3). The variants were located in mitochondrial tRNA genes (*MT‐TL1*, *MT‐TS1*, and *MT‐TK*), *MT‐ATP6* and complex I subunit genes (*MT‐ND4* and *MT‐ND5*). The range of mutant mtDNA levels in blood were as follows: mitochondrial tRNA genes (29–98%); *MT‐ATP6* (21–58%); and complex I subunit genes (28–73%). None of the clinical manifestations reported were typical for the well‐recognized phenotypes associated with the detected pathogenic mtDNA variants, and, in some instances, an alternative diagnosis was confirmed pathologically (subjects 15, 16, and 17) or genetically (subject 14). Furthermore, one patient (subject 12) had a clinicopathological diagnosis of inclusion body myositis, despite harboring 98% mutant levels of the pathogenic m.3243A>G variant in *MT‐TL1* associated with MELAS, and we are currently investigating the potential protective mechanisms preventing PMDs from manifesting in this individual.

### Pathogenic Mitochondrial DNA Variants That Exhibit Variable Clinical Penetrance

Pathogenic mtDNA variants that exhibit variable clinical penetrance were detected in 41 exomes. This group comprised mtDNA mutations associated with Leber hereditary optic neuropathy (LHON; n = 12), deafness and/or aminoglycoside‐induced deafness (n = 27), and maternally inherited cardiomyopathy (n = 2; see Supplementary Table S2). None of the patients (apart from subject 50) exhibited visual failure, deafness, or cardiomyopathy, despite many carrying near homoplasmic or homoplasmic mutant mtDNA levels. Subject 50 had bilateral sensorineural hearing loss associated with a complex neurological phenotype that would not be accounted for by the m.1555A>G mtDNA mutation alone. The absence of clinical manifestations, despite many patients harboring near homoplasmic or homoplasmic mutant mtDNA levels, is in keeping with the variable penetrance characteristic of these recurrent mtDNA variants.^14^

## Discussion

Re‐analysis of 11,424 exomes identified 24 unique pathogenic mtDNA variants across 64 exomes. Clinically significant pathogenic mtDNA variants were detected in 11 exomes from 9 unrelated families, thus confirming an mtDNA‐related PMD diagnosis in these patients. Of these 11 individuals, PMD had been considered in 6 (subjects 4–9), but mtDNA analysis was not completed prior to WES. This was partly due to the absence of characteristic mitochondrial abnormalities observed in muscle (subjects 6 and 7), but also the limited access to diagnostic mtDNA NGS at the local recruiting centers. In 5 of the 11 patients, a PMD diagnosis was not contemplated prior to WES despite phenotypes that, in hindsight, were compatible with the underlying pathogenic mtDNA variants subsequently detected. These findings highlight the major challenges faced by neurologists when evaluating patients with PMDs, which results from the clinical overlap that exists between PMD phenotypes and other neurological disorders. There has also been a shift toward a “genetics first” approach to diagnosing genetic neurological diseases, through the application of large nuclear gene panels and WES. However, mtDNA analysis is not routinely included in standard clinical WES bioinformatic pipelines, despite pathogenic mtDNA (rather than nuclear DNA) variants accounting for the majority of adult PMDs.^3^ Consequently, including mtDNA analysis when evaluating neurological disease exomes, represents an opportunity to capture genetically undiagnosed mtDNA‐related PMDs. Importantly, mtDNA NGS of a post‐mitotic tissue (eg, skeletal muscle) remains the “gold standard” molecular approach for excluding pathogenic mtDNA variants.

Muscle histopathology was available in 4 patients in whom PMD had been considered prior to WES; 2 patients (subjects 4 and 9) demonstrated typical pathological hallmarks of PMD (ragged red fibers), 1 patient exhibited a mild increase in lipid deposition only, albeit associated with reduced mitochondrial respiratory chain complex I enzyme activity (subject 6), and 1 patient was normal (subject 7). The most common brain magnetic resonance imaging (MRI) findings in patients with clinically significant pathogenic mtDNA variants included signal abnormalities of the basal ganglia and brainstem, in patients with Leigh/Leigh‐like syndrome (subjects 4, 7, 8, and 9) and MELAS (subject 6), and cerebellar atrophy (subjects 2, 4, and 5). Diffuse white matter changes were also observed in 2 patients (subjects 6 and 9).

Pathogenic mtDNA variants of undetermined clinical relevance were detected in 12 exomes from 12 unrelated families, whereas 41 exomes contained pathogenic mtDNA variants known to exhibit variable penetrance, despite near homoplasmic or homoplasmic mutant levels. The latter included pathogenic mtDNA variants associated with LHON (n = 12), deafness and/or aminoglycoside‐induced deafness (n = 27), and cardiomyopathy (n = 2). There was no evidence to suggest an underlying PMD in these individuals, and mutant mtDNA levels for some variants were below the threshold necessary for clinical symptoms to manifest (with the caveat that they were all detected in blood). However, there remains the possibility that disease onset could occur later in life or that subclinical manifestations are present (eg, cardiomyopathy or cardiac conduction defects). These findings have therefore been confirmed by diagnostic mtDNA NGS, with further analysis of a second tissue to assess risk, where appropriate. Reporting of secondary actionable findings (ie, genetic variants causing risk of an unrelated disease that would affect patient management) in large‐scale genetic testing is not straightforward, and mtDNA encoded genes are not currently included in the American College of Medical Genetics and Genomics (ACMG) medically actionable genes list.^15^, ^16^ However, the finding of a pathogenic mtDNA variant that potentially explains part or all of a clinical phenotype is within the remit of a primary finding, given it addresses the medical basis for requesting the test. There is no uniform definition of what is “medically actionable.”^17^ Consequently, when a pathogenic mtDNA variant did not clearly contribute toward the disease (see Table 3), the recruiting clinician was informed to determine whether it was actionable for that individual and whether the patient's consent supported reporting of secondary findings.

One limitation of mtDNA analysis using WES data is the depth of mtDNA coverage achieved. Unlike clinical diagnostic laboratories, which use dedicated deep sequencing of mtDNA,^18^ WES achieves comparatively low coverage of extracted mtDNA reads. Consequently, low‐level heteroplasmic mtDNA variants, particularly in blood, might remain undetected, despite the presence of higher, clinically relevant levels in post‐mitotic tissues. A second potential limitation is the spurious identification of a variant within a nuclear pseudogene that closely resembles an mtDNA gene – a so called “nuclear mitochondrial DNA segments” or “NUMTs.”^19^ However, we simultaneously aligned reads to nuclear and mitochondrial genomes and confirmed pathogenic mtDNA variants by sequencing long‐range PCR enriched mtDNA, thus the risk of such errors is extremely low.^20^

In conclusion, exome re‐analysis for pathogenic mtDNA variants in our cohort achieved a diagnostic uplift in 11 patients, thus highlighting the importance of including mtDNA analysis during the evaluation of neurological disease exomes.

## Author Contributions

O.V.P., D.M., E.B., C.E.W., J.V., M.G.H., A.P., and R.D.S.P. contributed to study concept and design. O.V.P., C.P., D.M., M.F., W.L.M., E.B., C.E.W., R.L., S.E., V.S., V.C., R.K., R.M., A.A.A., A.G., S.J.K., H.J., N.W., H.H., J.V., M.G.H., A.P., and R.D.S.P. contributed to data acquisition and analysis. O.V.P., C.P., D.M., M.F., W.L.M., E.B., J.V., M.G.H., A.P., and R.D.S.P. drafted the text and figures.

The members of the SYNaPS Study Group are included in the Supplementary online Table S3.

## Potential Conflicts of Interest

The authors declared no conflicts of interest.

## Supporting information


**Supplementary Table S1**: Recurrent pathogenic mitochondrial DNA variants that exhibit variable clinical penetrance at near homoplasmic or homoplasmic mutant levels

**Supplementary Table S2**: Pathogenic mitochondrial DNA variants that exhibit variable clinical penetrance at near homoplasmic or homoplasmic mutant levels detected in the 11,424 exomes analyzed

**Supplementary Table S3**: Members of the SYNaPS Study Group

## References

[1] Macken WL , Vandrovcova J , Hanna MG , Pitceathly RDS . Applying genomics and trascnritpomic advances to mitochondrial medicine. Nat Rev Neurol 2021. 10.1038/s41582-021-00455-2. Online ahead of print. 33623159

[2] Liu P , Meng L , Normand EA , et al. Reanalysis of clinical exome sequencing data. N Engl J Med 2019;380:2478–2480. 31216405 10.1056/NEJMc1812033 PMC6934160

[3] Gorman GS , Schaefer AM , Ng Y , et al. Prevalence of nuclear and mitochondrial DNA mutations related to adult mitochondrial disease. Ann Neurol 2015;77:753–759. 25652200 10.1002/ana.24362 PMC4737121

[4] Stenton SL , Prokisch H . Advancing genomic approaches to the molecular diagnosis of mitochondrial disease. Essays Biochem 2018;62:399–408. 29950319 10.1042/EBC20170110

[5] Van der Auwera GA , Carneiro MO , Hartl C , et al. From FastQ data to high confidence variant calls: the Genome Analysis Toolkit best practices pipeline. Curr Protoc Bioinformatics 2013;43:11.0.1–11.10.33. 25431634 10.1002/0471250953.bi1110s43 PMC4243306

[6] Benjamin D , Sato T , Cibulskis K , et al. Calling somatic SNVs and indels with Mutect2. *bioRxiv*. 2019.

[7] World Health Organization . International Statistical Classification of Diseases and Related Health Problems. Tenth Revision. Vol. 1: Tabular List (1992); Vol. 2: Instruction Manual (1993); Vol. 3: Index (1994). Geneva: WHO, 1992.

[8] McLaren W , Pritchard B , Rios D , et al. Deriving the consequences of genomic variants with the Ensembl API and SNP effect predictor. Bioinformatics 2010;26:2069–2070. 20562413 10.1093/bioinformatics/btq330 PMC2916720

[9] Brandon MC , Lott MT , Nguyen KC , et al. MITOMAP: a human mitochondrial genome database—2004 update. Nucleic Acids Res 2005;33:D611–D613. 15608272 10.1093/nar/gki079 PMC540033

[10] Thorburn DR , Rahman J , Rahman S . Mitochondrial DNA‐associated Leigh syndrome and NARP. In: Adam MP , Ardinger HH , Pagon RA , et al., eds. Seattle, WA: GeneReviews((R)), 1993. 20301352

[11] De Meirleir L , Seneca S , Lissens W , et al. Bilateral striatal necrosis with a novel point mutation in the mitochondrial ATPase 6 gene. Pediatr Neurol 1995;13:242–246. 8554662 10.1016/0887-8994(95)00184-h

[12] Honzik T , Tesarova M , Vinsova K , et al. Different laboratory and muscle biopsy findings in a family with an m.8851T>C mutation in the mitochondrial MTATP6 gene. Mol Genet Metab 2013;108:102–105. 23206802 10.1016/j.ymgme.2012.11.002

[13] Ng YS , Lax NZ , Maddison P , et al. MT‐ND5 mutation exhibits highly variable neurological manifestations at low mutant load. EBioMedicine 2018;30:86–93. 29506874 10.1016/j.ebiom.2018.02.010 PMC5952215

[14] Giordano C , Iommarini L , Giordano L , et al. Efficient mitochondrial biogenesis drives incomplete penetrance in Leber's hereditary optic neuropathy. Brain 2014;137:335–353. 24369379 10.1093/brain/awt343 PMC3914475

[15] Kalia SS , Adelman K , Bale SJ , et al. Recommendations for reporting of secondary findings in clinical exome and genome sequencing, 2016 update (ACMG SF v2.0): a policy statement of the American College of Medical Genetics and Genomics. Genet Med 2017;19:249–255. 27854360 10.1038/gim.2016.190

[16] Saelaert M , Mertes H , De Baere E , Devisch I . Incidental or secondary findings: an integrative and patient‐inclusive approach to the current debate. Eur J Hum Genet 2018;26:1424–1431. 29970927 10.1038/s41431-018-0200-9 PMC6138744

[17] Royal College of Physicians, Royal College of Pathologists and British Society for Genetic Medicine . Consent and Confidentiality in Genomic Medicine: Guidance on the Use of Genetic and Genomic Information in the Clinic. Report of the Joint Committee on Genomics in Medicine. 3rd ed. RCP, RCPath and BSGM: London, 2019.

[18] Zhang W , Cui H , Wong LJ . Comprehensive one‐step molecular analyses of mitochondrial genome by massively parallel sequencing. Clin Chem 2012;58:1322–1331. 22777720 10.1373/clinchem.2011.181438

[19] Samuels DC , Han L , Li J , et al. Finding the lost treasures in exome sequencing data. Trends Genet 2013;29:593–599. 23972387 10.1016/j.tig.2013.07.006 PMC3926691

[20] Santibanez‐Koref M , Griffin H , Turnbull DM , et al. Assessing mitochondrial heteroplasmy using next generation sequencing: a note of caution. Mitochondrion 2019;46:302–306. 30098421 10.1016/j.mito.2018.08.003 PMC6509278

