# Supplementary material for: Mitochondrial D NA Analysis from Exome Sequencing Data Improves Diagnostic Yield in Neurological Diseases
Source: Ann Neurol. 2021 Apr 1;89(6):1240–7. doi: 10.1002/ana.26063 (PMC8494076; doi:10.1002/ana.26063)
Supplement: Supplementary file 1 — Supplementary Table S1: Recurrent pathogenic mitochondrial DNA variants that exhibit variable clinical penetrance at near homoplasmic or homoplasmic mutant levels [file ANA-89-1240-s003.docx]

**Supplementary Table 1:** Recurrent pathogenic mitochondrial DNA variants that exhibit variable clinical penetrance at near homoplasmic or homoplasmic mutant levels

| **Mitochondrial DNA variant** | **Reported associated disease*** |
| --- | --- |
| m.1494C>T | Mitochondrial nonsyndromic hearing loss |
| m.1555A>G | Mitochondrial nonsyndromic hearing loss |
| m.3376G>A | LHON (“rare” pathogenic variant) |
| m.3460G>A | LHON (“common” pathogenic variant) |
| m.3635G>A | LHON (“rare” pathogenic variant) |
| m.3697G>A | LHON (“rare” pathogenic variant) |
| m.3700G>A | LHON (“rare” pathogenic variant) |
| m.3733G>A | LHON (“rare” pathogenic variant) |
| m.4171C>A | LHON (“rare” pathogenic variant) |
| m.4300A>G | MICM |
| m.10197G>A | LHON (“rare” pathogenic variant) |
| m.10663T>C | LHON (“rare” pathogenic variant) |
| m.11778G>A | LHON (“common” pathogenic variant) |
| m.13051G>A | LHON (“rare” pathogenic variant) |
| m.13094T>C | LHON (“rare” pathogenic variant) |
| m.14459G>A | LHON (“rare” pathogenic variant) |
| m.14482C>A/G | LHON (“rare” pathogenic variant) |
| m.14484T>C | LHON (“common” pathogenic variant) |
| m.14495A>G | LHON (“rare” pathogenic variant) |
| m.14502T>C | LHON (“rare” pathogenic variant) |
| m.14568C>T | LHON (“rare” pathogenic variant) |
| m.14674T>C/G | Transient infantile mitochondrial myopathy |

Abbreviations: LHON, Leber hereditary optic neuropathy; MICM, maternally inherited cardiomyopathy. *Brandon MC, Lott MT, Nguyen KC, et al. MITOMAP: a human mitochondrial genome database--2004 update. Nucleic Acids Res. 2005 Jan 1;33(Database issue):D611-3.
