# Supplementary material for: Mitochondrial D NA Analysis from Exome Sequencing Data Improves Diagnostic Yield in Neurological Diseases
Source: Ann Neurol. 2021 Apr 1;89(6):1240–7. doi: 10.1002/ana.26063 (PMC8494076; doi:10.1002/ana.26063)
Supplement: Supplementary file 2 — Supplementary Table S2: Pathogenic mitochondrial DNA variants that exhibit variable clinical penetrance at near homoplasmic or homoplasmic mutant levels detected in the 11,424 exomes analyzed [file ANA-89-1240-s002.docx]

**Supplementary Table 2:** Pathogenic mitochondrial DNA variants that exhibit variable clinical penetrance at near homoplasmic or homoplasmic mutant levels detected in the 11,424 exomes analyzed

| **Subject** | **Age (years) / Sex** | **mtDNA variant** | **Gene** | **No. Ref Reads** | **No. Alt Reads** | **Het (%)** | **Reported associated disease*** | **Subject phenotype / Family history** |
| --- | --- | --- | --- | --- | --- | --- | --- | --- |
| 24 | 27/m | m.1494C>T | *MT-RNR1* | 0 | 42 | 98 | DEAF | Cluster headache |
| 25 | 62/m | m.1555A>G | *MT-RNR1* | 18 | 3 | 14 | DEAF; ASID | Unaffected subject |
| 26 | 18/NA | m.1555A>G | *MT-RNR1* | 0 | 54 | 100 | DEAF; ASID | Dystonia |
| 27 | 70/NA | m.1555A>G | *MT-RNR1* | 0 | 13 | 100 | DEAF; ASID | Dystonia |
| 28 | 27/NA | m.1555A>G | *MT-RNR1* | 0 | 10 | 100 | DEAF; ASID | Dystonia |
| 29 | NA/m | m.1555A>G | *MT-RNR1* | 0 | 86 | 97 | DEAF; ASID | Microcephaly, GDD, seizures  Brother of Subject 30 |
| 30 | NA/f | m.1555A>G | *MT-RNR1* | 0 | 51 | 99 | DEAF; ASID | Microcephaly, GDD, seizures  Sister of Subject 29 |
| 31 | NA/f | m.1555A>G | *MT-RNR1* | 0 | 74 | 99 | DEAF; ASID | Unaffected mother of Subject 29 and 30 |
| 32 | NA | m.1555A>G | *MT-RNR1* | 0 | 25 | 98 | DEAF; ASID | PD |
| 33 | NA | m.1555A>G | *MT-RNR1* | 1 | 33 | 97 | DEAF; ASID | PD |
| 34 | NA/m | m.1555A>G | *MT-RNR1* | 3 | 447 | 99 | DEAF; ASID | FTD-MND |
| 35 | NA | m.1555A>G | *MT-RNR1* | 25 | 5 | 17 | DEAF; ASID | FTD |
| 36 | NA/m | m.1555A>G | *MT-RNR1* | 17 | 11 | 39 | DEAF; ASID | FTD |
| 37 | NA/f | m.1555A>G | *MT-RNR1* | 44 | 14 | 24 | DEAF; ASID | bvFTD |
| 38 | NA/f | m.1555A>G | *MT-RNR1* | 0 | 42 | 99 | DEAF; ASID | Neurogastrointestinal encephalopathy-like symptoms |
| 39 | NA | m.1555A>G | *MT-RNR1* | 0 | 85 | 98 | DEAF; ASID | MSA |
| 40 | NA | m.1555A>G | *MT-RNR1* | 50 | 19 | 28 | DEAF; ASID | MSA |
| 41 | NA | m.1555A>G | *MT-RNR1* | 1 | 99 | 95 | DEAF; ASID | MSA |
| 42 | NA | m.1555A>G | *MT-RNR1* | 0 | 11 | 92 | DEAF; ASID | PD |
| 43 | NA | m.1555A>G | *MT-RNR1* | 0 | 17 | 97 | DEAF; ASID | PD |
| 44 | NA | m.1555A>G | *MT-RNR1* | 6 | 27 | 81 | DEAF; ASID | Unspecified neurological presentation |
| 45 | NA | m.1555A>G | *MT-RNR1* | 0 | 18 | 93 | DEAF; ASID | Cluster headache |
| 46 | NA | m.1555A>G | *MT-RNR1* | 1 | 17 | 94 | DEAF; ASID | EOEE |
| 47 | NA | m.1555A>G | *MT-RNR1* | 10 | 8 | 44 | DEAF; ASID | EOEE |
| 48 | NA | m.1555A>G | *MT-RNR1* | 0 | 31 | 97 | DEAF; ASID | NBIA |
| 49 | NA/m | m.1555A>G | *MT-RNR1* | 0 | 35 | 96 | DEAF; ASID | Unaffected subject |
| 50 | 5/m | m.1555A>G | *MT-RNR1* | 0 | 18 | 100 | DEAF; ASID | GDD, cerebellar syndrome, generalized chorea, neuropathy, short stature, SNHL |
| 51 | NA | m.3460G>A | *MT-ND1* | 30 | 12 | 29 | LHON | PSP |
| 52 | NA | m.3460G>A | *MT-ND1* | 1 | 10 | 88 | LHON | Unaffected subject |
| 53 | NA | m.3460G>A | *MT-ND1* | 0 | 52 | 99 | LHON | Unaffected subject |
| 54 | NA | m.4300A>G | *MT-TI* | 25 | 17 | 40 | MICM | Unaffected child of Subject 55 |
| 55 | NA/f | m.4300A>G | *MT-TI* | 12 | 4 | 25 | MICM | Epileptic seizures  Mother of subject 54 |
| 56 | NA | m.14484T>C | *MT-ND6* | 0 | 20 | 100 | LHON | Unspecified neurological presentation |
| 57 | NA | m.14484T>C | *MT-ND6* | 0 | 14 | 97 | LHON | Unspecified neurological presentation |
| 58 | NA | m.14484T>C | *MT-ND6* | 0 | 12 | 100 | LHON | Unspecified neurological presentation |
| 59 | NA | m.14484T>C | *MT-ND6* | 0 | 11 | 100 | LHON | PD |
| 60 | 24/m | m.14484T>C | *MT-ND6* | 0 | 22 | 100 | LHON | Sporadic demyelinating sensory-motor neuropathy, onset at 2.5 years |
| 61 | NA | m.14484T>C | *MT-ND6* | 0 | 38 | 99 | LHON | NBIA |
| 62 | 51/m | m.14484T>C | *MT-ND6* | 0 | 27 | 98 | LHON | PD |
| 63 | 51/m | m.14484T>C | *MT-ND6* | 0 | 11 | 100 | LHON | PD |
| 64 | NA | m.14484T>C | *MT-ND6* | 0 | 19 | 100 | LHON | Cluster headache |

Abbreviations: ASID, autism spectrum intellectual disability; DEAF, sensorineural Deafness; EOEE, early onset epileptic encephalopathy; FTD, frontotemporal dementia; bvFTD, behavioral variant FTD; FTD-MND, motor neuron disease and FTD; GDD, global developmental delay; Het, heteroplasmy; LHON, Leber hereditary optic neuropathy; MICM, maternally inherited cardiomyopathy; MSA, multi-system atrophy; mtDNA, mitochondrial DNA; NA, not available; NBIA, neurodegeneration with brain iron accumulation; No., number; PD, Parkinson’s disease; PSP, progressive supranuclear palsy; SNHL, sensorineural hearing loss. *Brandon MC, Lott MT, Nguyen KC, et al. MITOMAP: a human mitochondrial genome database--2004 update. Nucleic Acids Res. 2005 Jan 1;33(Database issue):D611-3.
