# Supplementary material for: Mitochondrial D NA Analysis from Exome Sequencing Data Improves Diagnostic Yield in Neurological Diseases
Source: Ann Neurol. 2021 Apr 1;89(6):1240–7. doi: 10.1002/ana.26063 (PMC8494076; doi:10.1002/ana.26063)
Supplement: Supplementary file 3 — Supplementary Table S3: Members of the SYNaPS Study Group [file ANA-89-1240-s001.docx]

**Supplementary Table 3:** Members of the SYNaPS Study Group

| **Name** | **Affiliation** |
| --- | --- |
| Issam Alkhawaja MD, MSc | Pediatric Neurology Unit, Al Bashir Hospital, Amman, Jordan |
| Selina Banu MD | Department of Pediatric Neurology, ICH and SSF Hospital Mirpur, Dhaka, 1216, Bangladesh |
| Maria Bonsignore MD | Child Neuropsychiatry Unit, Department of Human Pathology of the Adult and Developmental Age, University Hospital "G. Martino", Messina, Italy |
| Marianthi Breza MD | 1st Department of Neurology, Eginition Hospital, Medical School, National and Kapodistrian University of Athens, Athens, Greece |
| Gabriella Di Rosa MD | Child Neuropsychiatry Unit, Department of Human Pathology of the Adult and Developmental Age, University Hospital "G. Martino", Messina, Italy |
| Morteza Heidari MD | Department of Pediatric Neurology, Children's Medical Center, Pediatric Center of Excellence, Tehran, Iran |
| Georgios Koutsis MB BChir, PhD | 1st Department of Neurology, Eginition Hospital, Medical School, National and Kapodistrian University of Athens, Athens, Greece |
| Arn M.J.M. van den Maagdenberg MD | Department of Neurology, Leiden University Medical Center, Leiden, Netherlands |
| Alfons Macaya MD, PhD | Pediatric Neurology Section, Hospital Universitari Vall d'Hebron, Barcelona, Spain |
| Alexander Münchau MD | Institute of Systems Motor Science, University of Lübeck, Lübeck, Germany |
| Carmela Scuderi MD | Unit of Neuromuscular Diseases, Oasi Research Institute-IRCCS, Troina, Italy |
| Nazira Zharkinbekova MD | South Kazakhstan Medical Academy, Department of Neurology, Shymkent, Kazakhstan |
